# Follicular Proinflammatory Cytokines and Chemokines as Markers of IVF Success

**DOI:** 10.1155/2012/606459

**Published:** 2011-10-05

**Authors:** Aili Sarapik, Agne Velthut, Kadri Haller-Kikkatalo, Gilbert C. Faure, Marie-Christine Béné, Marcelo de Carvalho Bittencourt, Frédéric Massin, Raivo Uibo, Andres Salumets

**Affiliations:** ^1^Department of Immunology, Institute of General and Molecular Pathology, University of Tartu, Ravila 19, Tartu 50411, Estonia; ^2^Institute of Gene Technology, Tallinn University of Technology, Akadeemia Street 15, Tallinn 12618, Estonia; ^3^Competence Centre on Reproductive Medicine and Biology, Tiigi 61b, Tartu 50410, Estonia; ^4^Department of Obstetrics and Gynecology, University of Tartu, L. Puusepa 8, 51014 Tartu, Estonia; ^5^Women's Clinic, Tartu University Hospital, L. Puusepa Street 1a, 50406 Tartu, Estonia; ^6^Laboratoire d'Immunologie, Université Henri Poincaré, BP 184, Vandoeuvre lès Nancy, 54500 Nancy, France

## Abstract

Cytokines are key modulators of the immune system and also contribute to regulation of the ovarian cycle. In this study, Bender MedSystems FlowCytomix technology was used to analyze follicular cytokines (proinflammatory: IL-1**β**, IL-6, IL-18, IFN-**γ**, IFN-**α**, TNF-**α**, IL-12, and IL-23;, and anti-inflammatory: G-CSF), chemokines (MIP-1**α**, MIP-1**β**, MCP-1, RANTES, and IL-8), and other biomarkers (sAPO-1/Fas, CD44(v6)) in 153 women undergoing *in vitro* fertilization (IVF). Cytokine origin was studied by mRNA analysis of granulosa cells. Higher follicular MIP-1**α** and CD44(v6) were found to correlate with polycystic ovary syndrome, IL-23, INF-**γ**, and TNF-**α** with endometriosis, higher CD44(v6) but lower IL-**β** and INF-**α** correlated with tubal factor infertility, and lower levels of IL-18 and CD44(v6) characterized unexplained infertility. IL-12 positively correlated with oocyte fertilization and embryo development, while increased IL-18, IL-8, and MIP-1**β** were associated with successful IVF-induced pregnancy.

## 1. Introduction

Immunological abnormalities have been implicated in female reproductive failure, but whether these represent a cause or effect is unknown [1, 2]. According to our previous research, cellular and, particularly, humoral autoimmunologic perturbations are responsible for development of female infertility. Disturbances in the humoral immune system may lead to impairments in ovarian folliculogenesis [3–5], a long and complex process in which both the endocrine and immune systems play significant roles. 

Cytokines, originally identified as products of immune cells, are important mediators of immune responses. These proteins are able to stimulate or inhibit cell growth, regulate cell differentiation, induce cell chemotaxis, and modulate the expression of other cytokines. However, recent research has indicated that cytokines are synthesized by a broad range of nonimmune cell types, including the normal ovarian cells. Cytokine function in the ovary has been described as promoting processes of follicular growth, steroidogenesis, recruitment and activation of leukocytes necessary for ovulation and tissue remodelling during ovulation, luteinization, and luteolysis [6]. 

To gain a more detailed understanding of the cytokines involved in female fertility and their role in pregnancy outcome, we assessed 16 different follicular cytokines during infertility treatment. In particular, we evaluated the cytokines for Th1/proinflammation (interleukin- (IL-) 1*β*, IL-6, IL-12, IL-18, IL-23, interferon (IFN)-*γ*, IFN-*α*, and tumor necrosis factor-(TNF-) *α*) and anti-inflammation (granulocyte colony stimulating factor (G-CSF)), the principal chemokines (macrophage inflammatory protein- (MIP-) 1*α*, MIP-1*β*, monocyte chemotactic protein- (MCP-) 1, regulated on activation, normal T expressed and secreted (RANTES) and IL-8), and other biomarkers (soluble apoptosis antigen (sAPO)-1/Fas and CD44 variant isoform CD44(v6)) secreted into the follicular fluid. The cytokines chosen for evaluation were shown in our previous study to be appreciably expressed in follicular granulosa cells at the mRNA level [7]; moreover, the importance of these particular cytokines in ovarian function has been proposed by others [8].

IL-1*β*, IL-6, and IL-18 are key mediators of inflammation and mediate many pathways of the normal immune response [9–11]. Human IFN-*α* comprises a family of extracellular signalling proteins with demonstrated antiviral, antiproliferating, and immunomodulatory activities [12]. The type II interferon IFN-*γ* is another proinflammatory cytokine and has been implicated in the development of a variety of autoimmune diseases [13]. IL-12 regulates cell-mediated immune responses. The p40 subunit of IL-12 is shared with IL-23 and is essential for recruitment and activation of many inflammatory cell types. Both of these cytokines interact with the innate and adaptive immune systems [14]. TNF-*α*, an acute phase protein, is critically involved in innate immune responses caused by pathogen exposure [15] but can also mediate noninfectious inflammatory processes such as autoimmunity and cancer [16]. 

Among the chemokines examined in this study, IL-8 is a neutrophil-specific factor involved in inflammatory processes and angiogenesis [17]. MIP-1*α* and MIP-1*β* are known as CC chemokines, and both act as chemoattractants for T cells and monocytes to mediate beneficial inflammatory processes, such as wound healing [18]. Meanwhile, MCP-1 and RANTES are potent chemoattractants of monocytes and T lymphocytes [19]. 

The other biomarkers examined in this study are established immunomodulators. G-CSF acts as a growth factor for haematopoietic cells [20]. APO-1 regulates tissue homeostasis by acting as the receptor for Fas ligand, the binding of which triggers a signaling cascade that leads to apoptosis inhibition [21]. And CD44(v6), a splice variant of the CD44, is a transmembrane glycoprotein associated with cell adhesion and has mostly been investigated in tumours [22]. 

In recent decades, above-mentioned cytokines have become the subject of studies examining normal mammalian reproduction [23], which have indicated a significant role for these factors in supporting female fertility. Thus, we carried out a simultaneous (multiplex) examination of these cytokines and biomarkers in follicular fluid of infertile women in order to assess their effects on oocyte and embryo quality and on pregnancy outcome of *in vitro* fertilization (IVF) treatment. The approach of cytokine profiling using multiplex assays offers a promising tool for identifying condition-specific biomarker panels with high accuracy [24]. We employed the Bender MedSystems FlowCytomix platform, which uses antibody-coated autofluorescent beads to simultaneously measure corresponding analytes from small sample volumes and low concentrations [24], facilitating time- and cost-efficient high-throughput screening. In addition, we sought to determine the origin of the secreted cytokines by performing mRNA analysis from two distinct follicular somatic cell populations: mural and cumulus granulosa cells (MGC and CGC, resp.).

## 2. Materials and Methods

### 2.1. Patients

The Ethics Committee on Human Research of the University of Tartu approved this study, and informed consent was obtained from all patients. The study group consisted of 153 women, aged 33.3 ± 4.5 years (mean ± standard deviation), who underwent IVF at Nova Vita Clinic between 2007 and 2010. IVF with intracytoplasmic sperm injection (ICSI) was performed in all women in this cohort, and the case was either male factor infertility or previous oocyte fertilization failure. The causes of infertility were distributed as follows: male factor infertility (43.8%, *n* = 67), tubal factor infertility (TFI; 28.8%, *n* = 44), polycystic ovary syndrome (PCOS; 5.2%, *n* = 8), endometriosis (15.0%, *n* = 23), unexplained infertility (4.6%, *n* = 7), and other reasons (2.6%, *n* = 4). 

Ovarian hormonal stimulation was conducted according to a protocol of gonadotrophin-releasing hormone (GnRH) antagonist (Cetrotide; Merck Serono, Geneva, Switzerland) administered with recombinant follicle-stimulating hormone (Gonal-F; Merck Serono or Puregon, Schering-Plough, Kenilworth, NJ, USA). ICSI was performed at 4–6 h after oocyte pickup (OPU), resulting in a 68.9% fertilization rate. ICSI-derived embryos were cultured up to 48 h, after which good-quality embryos were identified by the presence of at least four blastomeres and ≤20% fragmentation. The rate of good-quality embryos was calculated as the proportion (%) of good-quality embryos out of all fertilized oocytes. Two embryos were chosen for transfer to the uterus, and 25.5% of clinical pregnancies resulted per embryo transfer. Clinical confirmation of intrauterine pregnancy was made using an ultrasound scan performed at the 6th or 7th week after transference. Follicular fluid samples from each individual were taken from a single follicle on the day of OPU and stored at −80°C until further use.

### 2.2. Flow Cytometry Analysis

Altogether, 16 biomarkers (divided into two 8 plexes) were evaluated from the individual follicular fluid samples by using a commercially available FlowCytomix Human Basic Kit Assay (Bender MedSystems, Vienna, Austria) and following the manufacturer's instructions. Quantitation measurements were performed by flow cytometer instrument FC 500 and accompanying CXP Software (Beckman Coulter, Calif, USA). The first 8 plex consisted of: IL-23, sAPO-1/Fas, MIP-1*β*, MIP-1*α*, CD44(v6), IL-8, G-CSF, and RANTES. The second 8-plex consisted of IL-12p70, IFN-*γ*, MCP-1, IL-6, IFN-*α*, IL-18, Il-1*β*, and TNF-*α*. Samples were prepared for processing by first thawing follicular fluids and centrifuging the whole volume at 450 g for 10 min. The resulting supernatants were used for analysis. FlowCytomix Pro 2.3 Software was used to perform calculations (Bender MedSystems). Standard curves for each biomarker were generated with manufacturer-supplied reference analyte (pg/mL concentrations). The concentration of a biomarker was calculated as mean fluorescent intensity divided by single median standard curve.

### 2.3. Gene Expression Analysis

Gene expression studies of the measured cytokines were performed by real-time PCR of mRNA isolated from MGC and CGC from six patients. MGC were obtained from follicular fluid after OPU, and CGC were collected 4 h after OPU during oocyte denudation with bovine type IV-S hyaluronidase (Sigma-Aldrich, St-Louis, Mo, USA). The detailed isolation protocol has been previously published [7]. For leukocyte elimination, the MGC pool was incubated with CD45-coated magnetic beads (Dynabeads; Invitrogen, Oslo, Norway) for an additional 1 h at 4°C, followed by magnet-based cell sorting (DynaMag-15; Invitrogen) according to the manufacturer's protocol. Total RNA was extracted, and real-time PCR analysis was performed using either commercially available real-time PCR arrays (products PAHS-011A and PAHS-021A; SABiosciences, Frederick, Md, USA) or in-house designed and synthesized primers when the desired transcripts were not included in the kits or the quality of amplification, and melting curves were not satisfactory. Primers for Fas were designed to exclusively detect the soluble isoform, and those for CD44 were designed to amplify only exon 11 (the variable region 6). All primer sequences used in this study are listed in Table 1. 

For double-stranded cDNA synthesis, 1 *μ*g of high-quality total RNA was treated with DNase (Fermentas, Burlington, ON, Canada) and reverse transcribed to cDNA using the RT^2^ First Strand Kit (SABiosciences) according to the manufacturer's protocols. RT² SYBR Green/ROX qPCR Master Mix (SABiosciences) and cDNA template were added to the array and product amplification was performed on a 7500 real time PCR System (Applied Biosystems, Foster City, Calif, USA). Those real-time PCR reactions using in-house primers were performed using the Power SYBR Green PCR Master Mix (Applied Biosystems) and the 7900HT real-time PCR instrument (Applied Biosystems).

Results were analyzed with instrument-specific software using the ΔΔCt relative quantification method. Three housekeeping genes were used for normalization of the amplification data: beta actin, glyceraldehyde-3-phosphate dehydrogenase, and ribosomal protein RPL13A.

### 2.4. Statistical Analysis

The R2.3.1 A Language and Environment (Free Software Foundation, Boston, Mass, USA) was used to perform *t*-, Mann-Whitney *U*- and proportion tests and adjusted simple regression analysis. A *P* value <0.05 was considered as indicative of statistical significance.

## 3. Results

**Figure 1:**
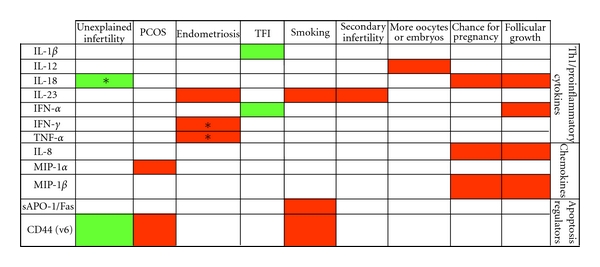
Associations between biomarkers and infertility parameters. Red boxes indicate positive association, green boxes negative association; empty boxes indicate no association found by adjusted regression analysis. Male factor infertility was chosen as a reference group, but in cases marked with ∗. TFI was used as a reference. Abbreviations are as mentioned in the text.

Table 2 summarizes the clinical data and infertility treatment parameters, while Table 3 lists detected concentrations of the tested biomarkers.  Figure 1 summarizes the main associations observed between the clinical data and the levels of follicular biomarkers, according to analysis by adjusted regression models.

### 3.1. Associations between Infertility Cause and Levels of Biomarkers in Follicular Fluid

Patients characterized by male factor infertility represented the reference group in simple regression analysis, unless otherwise stated. Our results indicated that women with TFI had lower concentrations of IL-1*β*-adjusted *r* = −12.6 pg/mL, *P* = 0.037), and lower IFN-*α* levels were also significantly associated with TFI when the status of current smoking was included in the model (adjusted *r* = −13.9 pg/mL, *P* = 0.046). 

PCOS patients were characterized by significantly higher follicular levels of CD44(v6) (age-adjusted *r* = 2072.7 pg/mL, *P* = 0.010) and MIP-1*α* (adjusted for age, cause of infertility, and follicular count in prestimulatory ovary *r* = 3111.7 pg/mL, *P* = 0.007). Women with endometriosis presented with higher levels of IL-23 than male factor infertility patients (adjusted by follicular number prior to stimulation *r* = 157.1 pg/mL, *P* = 0.025). Moreover, when compared to TFI patients, women with endometriosis had higher levels of follicular TNF-*α* (age-adjusted *r* = 2.6 pg/mL, *P* = 0.047) and IFN-*γ* (adjusted by follicular number prior to stimulation *r* = 16.4 pg/mL, *P* = 0.030). In women with unexplained infertility, significantly lower levels of follicular CD44(v6) were measured, as compared to male factor infertility patients (age-adjusted *r* = −1888.4 pg/mL, *P* = 0.025). However, when compared to TFI patients, the unexplained infertility patients also had lower levels of IL-18 (age-adjusted *r* = −186.7 pg/mL, *P* = 0.021). 

Active smoking was associated with elevated follicular CD44(v6) levels (adjusted for age and cause of infertility *r* = 1227.8 pg/mL, *P* = 0.019 versus never smokers group) and sAPO-1/Fas levels (adjusted *r* = 464.9 pg/mL, *P* = 0.031 versus never-smokers group). Similarly, follicular IL-23 levels were higher in women who reported history of smoking or current smoking, as compared to never-smokers, regardless of age or cause of infertility (adjusted *r* = 107.6 pg/mL, *P* = 0.043). In addition, an elevated IL-23 concentration was associated with women experiencing secondary infertility rather than primary infertility (regardless of the cause of infertility; adjusted *r* = 94.6 pg/mL, *P* = 0.043).

### 3.2. Associations between Infertility Treatment Parameters and Biomarker Levels in Follicular Fluid

A positive association was determined to exist between the concentration of follicular IL-12 and the number of fertilized oocytes (adjusted *r* = 0.15 pg/mL per every additional 2PN oocyte, *P* = 0.007) and the proportion of good-quality embryos (adjusted *r* = 0.22 pg/mL per every additional embryo, *P* = 0.006), when the data were adjusted for age, cause of infertility, and follicular size. Achieving intrauterine pregnancy was associated with higher levels of follicular MIP-1*β*, as compared to hCG-negative patients (adjusted for age and cause of infertility *r* = 48.0 pg/mL, *P* = 0.047). In addition, follicular MIP-1*β* and IFN-*α* levels were both positively associated with the diameter of a follicle (adjusted *r* = 7.8 pg/mL, *P* = 0.037 and *r* = 2.4 pg/mL for every millimeter in diameter, *P* = 0.023, resp.), regardless of age or cause of infertility. 

The concentration of IL-8 in follicular fluid was positively associated with intrauterine pregnancy (adjusted for age, cause of infertility, rate of good-quality embryos transferred, and endometrial thickness *r* = 207.5 pg/mL, *P* = 0.051), and also with parity (adjusted for age and cause of infertility *r* = 150.6 pg/mL for every child born, *P* = 0.039). Not surprisingly, IL-8 was also associated with higher levels of serum progesterone after ovarian stimulation (adjusted *r* = 4.7 pg/mL, *P* = 0.031). 

Follicular IL-18 levels appeared to be positively correlated with several outcomes, including increased chance for intrauterine pregnancy (adjusted for the cause of infertility *r* = 71.6 pg/mL, as compared to hCG-negative patients, *P* = 0.054), number of fetuses detected by ultrasonography (adjusted for age, cause of infertility, number of embryos transferred, rate of good-quality embryos among them, and endometrial thickness *r* = 67.2 pg/mL for every additional fetus, *P* = 0.020), and with increased parity (adjusted for age and cause of infertility *r* = 60.7 pg/mL for every child to give birth, *P* = 0.038). Interestingly, the levels of both follicular IL-8 and IL-18 increased as follicles grew (adjusted for age and cause of infertility *r* = 40.2 pg/mL, *P* = 0.005, and *r* = 1  3.1 pg/mL for every additional millimeter in diameter, *P* = 0.022, resp.).

### 3.3. mRNA Analysis of the Measured Protein Transcripts from MGC and CGC

**Figure 2:**
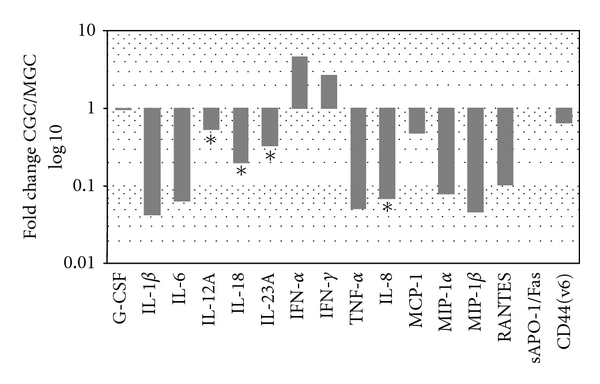
Differential expression of measured protein transcripts in cumulus and mural granulosa cells. ∗ Differences in expression were statistically significant. CGC: cumulus granulosa cells, MGC: mural granulosa cells.

Our mRNA expression analysis demonstrated that most of the studied transcripts were more abundantly expressed in MGC (Figure 2). G-CSF and sAPO-1/Fas were not differentially expressed in the two cell types. Both of the interferons examined were found to be more highly expressed in CGC, although this result was not statistically significant (Table 4). When the abundance of intracellular transcripts was analyzed, the mRNA levels were found to differ by several orders of magnitude and were characterized by substantial interpatient variability (Table 4).

## 4. Discussion

In the current study, we evaluated the expression of 16 different biomarkers in the follicular fluid of infertile women by using multiplex assay from Bender MedSystems. These biomarkers included Th1/proinflammatory and anti-inflammatory cytokines, chemokines and antiapoptotic biomarkers that had previously been implicated in ovarian function by our previous study [7]. Ultimately, we found that 12 of the 16 examined biomarkers were associated with a cause of infertility or IVF treatment outcome.

The mammalian ovulation event can be considered from the perspective of an inflammatory reaction, with proinflammatory cytokines produced and functionally interacting throughout the process [10]. For example, IL-1*β* has been evidenced to participate in ovulation induction by facilitating follicular rupture [27]. The fact that we found lower values of IL-1*β* in TFI patients indicates that impairment of folliculogenesis might have occurred and contributed to the infertility of these women. IL-18 is known to induce cytokines that are important for both folliculogenesis and ovulation, including IL-1*β*, TNF-*α*, and IFN-*γ* [28]. Our finding that levels of IL-18 were relatively low in unexplained infertility patients might then reflect an underlying perturbed immunological profile for this infertility cause. Importantly, IL-18 has been suggested to favor ovarian folliculogenesis; a positive correlation has been reported between the level of follicular IL-18 and the number of retrieved oocytes and implantation success in women with different etiologies of infertility [29, 30]. Our finding that follicular growth positively correlates with IL-18 levels indirectly supports the role of IL-18 in follicle maturation. Furthermore, our finding that higher follicular IL-18 was associated positively with parity indicates that this cytokine may increase the chance for pregnancy.

The elevated levels of follicular IFN-*γ* found in our group of endometriosis patients is in agreement with previous results obtained with serum samples [27]. Increased production of IFN-*γ* may reflect the immune system's efforts to overcome apoptosis inhibition and to decrease cell proliferation in the case of endometriosis [31]. Additionally, while a temporary increase in the concentration of IFN-*γ* seems to be essential for ovulation, IFN-*γ* levels that exceed normal physiologic concentrations may inhibit ovulation and contribute to early pregnancy loss [28]. IFN-*α* is synthesized primarily in response to infection, but the IFN-*α* signaling pathways have also been demonstrated to be involved in reproduction processes, even in the absence of detectable infection [12]. The fact that the group of healthy women (with male factor infertility) in our study possessed higher levels of IFN-*α* than did women with TFI further supports a positive role for IFN-*α* in reproduction. In addition, the positive correlation that was identified between follicular IFN-*α* levels and follicular diameter was in accordance with previous IFN-*α* data from preovulatory granulosa cells [32]. 

We observed elevated levels of TNF-*α* in endometriosis patients, as compared to TFI patients. It is possible that this finding simply reflects increased TNF-*α* serum levels that had infiltrated into the follicular fluid [33] or increased secretion by granulosa cells induced by the inflammatory pelvic milieu in endometriosis [34]. TFI patients' expression of follicular TNF-*α* has also been previously suggested to be below the threshold of standard detection systems [6]. TNF-*α* in IVF has already been the subject of much study by infertility researchers. Some authors have concluded that follicular TNF-*α* might deteriorate the microenvironment in the follicle, thereby negatively affecting oocyte and embryo quality [35]. Still others have proposed a positive role of TNF-*α* regarding oocyte quality, and ovulation [36]. Overall, the roles of TNF-*α* in female reproduction are likely to be complex and dynamically involved in the different stages of folliculogenesis [37].

Previous studies examining IL-12 in the follicular fluid have yielded contradictory results. Nevertheless, a majority of the findings have indicated that IL-12 is associated with a negative effect on folliculogenesis, oocyte quality and implantation [9, 20, 38]. We failed to detect any correlation between the follicular level of IL-12 and the pregnancy outcome of IVF. Nonetheless, there was a positive association identified between IL-12 and the quality of oocytes and embryos. Our results are similar to a study published by Ostanin et al. [39], wherein the authors reported that follicular concentration of IL-12 was elevated in women who produced more high-quality oocytes. IL-12 is a Th1 cytokine that can become cytotoxic at high levels. It is, therefore, not unexpected that high concentrations of IL-12 in the follicular fluid might impair the natural process of folliculogenesis and ovulation [38]. However, in the current study, the mean concentration of IL-12 was found to be more than 10-fold lower than that reported in studies that had concluded deleterious function of IL-12 on reproduction [9, 20, 38]. Thus, we suggest a dose-dependent role for IL-12 in the follicles. 

We also determined that endometriosis was associated with increased levels of follicular IL-23. Given that IL-23 is known to participate in autoimmune diseases by promoting inflammation, a hallmark of endometriosis, this result was not surprising. Impaired follicular fluid microenvironment characterized by elevated inflammatory cytokines may in fact be the cause for poor oocyte quality, which in turn could lead to poor IVF outcome in patients with endometriosis [19]. The detrimental effect of IL-23 on fecundity is further supported by our findings of higher levels of IL-23 in women who smoked or who suffered from secondary infertility. 

MIP-1*α* is a marker for ongoing acute or chronic inflammatory host responses [18, 40]. Dahm-Kähler et al. [41] failed to detect MIP-1*α* in follicular fluids of unstimulated menstrual cycles, leading to their conclusion that MIP-1*α* is not produced under physiological conditions. Our contradictory findings of elevated levels of MIP-1*α* in PCOS patients may reflect a character of increased inflammation in stressed ovaries. To date, very few studies have appeared in the literature that investigate the function of MIP-1*β* in female reproduction, although this chemokine has been suggested to promote folliculogenesis and pregnancy establishment [39]. Such a positive role was also supported by our finding that higher follicular fluid MIP-1*β* levels correlated with follicular growth and achieving pregnancy. 

The correlation of IL-8 concentration with follicular growth is in accordance with previously reported results. When taking into consideration that IL-8 has also been detected in unstimulated cycles [38], the involvement of this chemokine in the natural process of folliculogenesis and ovulation can be assumed [42]. Moreover, a recent study showed that lower serum levels of IL-8 correlated with a higher risk for extrauterine pregnancy [43]. Thus, our finding of higher follicular fluid IL-8 in cases of normal intrauterine pregnancy seems sensible. Nonetheless, two previous studies demonstrated no correlations between follicular fluid IL-8 concentration and IVF cycle parameters or pregnancy results [38, 42]. The discrepant results obtained from these studies and our own could be due to differences in sample sizes, patient groups examined, or detection methods used; this issue needs further investigation. 

sAPO-1/Fas mediates apoptosis inhibition, which is important in preventing oocytes from succumbing to atresia during follicular maturation [21]. Increased sAPO-1/Fas levels have also been associated with enhanced activity of smoking-induced antiapoptotic signaling pathways in the oral cavity, which leads to epithelial hyperplasia [44]. In our study, we detected higher levels of sAPO-1/Fas in active smokers. Thus, our findings suggest that a compensatory increase of sAPO-1/Fas was established in the apoptosis-favoured environment of the follicles in active smokers. A similar effect has also been proposed for CD44(v6) in the ovary, where macrophage membrane-expressed CD44 protein has been shown to participates in clearance of apoptotic granulosa cells [44, 45]. Our findings of lower levels of CD44(v6) in unexplained infertility and higher levels in PCOS and active smokers might reflect impaired apoptosis mechanisms in the ovaries of these patients. 

Considering that cytokines likely affect ovarian function, one could argue about the source of these immunomodulatory factors in follicular fluid. The ovulatory process is comparable to a classical local inflammatory reaction, and leukocytes have been shown to participate actively in the cyclic events of the ovary [6]. However, it is unlikely that migrating leukocytes producing proinflammatory cytokines represent the principal mechanism by which ovarian folliculogenesis is regulated [6]. Increased levels of serum-derived cytokines in follicular fluid have been demonstrated in endometriosis [33]. In addition, upregulated expression of proinflammatory cytokines by granulosa cells has been detected in cases of infertility [7]. Here, we confirmed our previous findings from the Affymetrix GeneChip platform using real-time PCR analysis to monitor mRNA expression in different conditions of infertility, as compared to levels expressed in conditions of normal fertility. To the best of our knowledge, our results represent the first description of the human granulosa cell expression profile of IL-12A, IL-23A, IL-18, MIP-1*α*, MIP-1ß, IFN-*α*, IFN-*γ*, and sAPO-1/FAS. MIP-1ß has been studied in the mouse cumulus-oocyte complex, where its expression increased in response to experimental exposure to hyaluronan fragments, and the related signal was determined to be mediated by Toll-like receptors [46]. On the other hand, luteinizing hormone induction of IFN-*α* was shown in rats and determined to function as a modulator of steroidogenesis and MGC differentiation [47]. It is well known that cytokines and apoptosis networks functionally interact with one another in a variety of mammalian, and human, tissues. Therefore, our results also indicate a strong role of these proteins in human follicular physiology.

In conclusion, we discovered that various infertility etiologies are accompanied by distinct intrafollicular cytokine profiles. Furthermore, some of the cytokines evaluated, such as IL-12, were determined to influence oocyte fertilization and embryo quality, while others, such as IL-18, IL-8, and MIP-1*β*, were found to be correlated with successful pregnancy following IVF treatment. Collectively, these factors appear to be promising prognostic markers for IVF success and should be evaluated as such by future prospective studies.

## Figures and Tables

**Table 1:** List of primers used for real-time PCR analysis.

Gene	Forward primer	Reverse primer	NCBI reference
G-CSF [25]	GCTTGAGCCAACTCCATAGC	CAGATGGTGGTGGCAAAGTC	NM_001178147.1, NM_172220.2, NM_172219.2, NM_000759.3
IL-23A [26]	TGTTCCCCATATCCAGTG	TCCTTTGCAAGCAGAACTGA	NM_016584.2
IFN-*γ*	TGATGGCTGAACTGTCGCCAGC	CTGGGATGCTCTTCGACCTCGA	NM_000619.2
MIP-1*α*	TCAGAAGGACACGGGCAGCAGA	TCAGCAGCAAGTGATGCAGAGAAC	NM_002983.2
sAPO-1/Fas	CCAAGTGCAAAGAGGAAGTGAAGAG	TGGTTTTCCTTTCTGTGCTTTCTGC	NM_152871.2
CD44(v6)	GCTACCACAGCCTCAGCTCA	ACCTCGTCCCATGGGGTGTGA	NA*

*The forward primer was designed to cross the junction between exons 5 and 11, characteristic for only CD44(v6)-soluble splice isoform not described in NCBI database.

**Table 2:** Clinical and IVF treatment parameters of patient groups.

	Male factor infertility (*N* = 67)^a^	Tubal factor infertility (*N* = 44)	Polycystic Ovary Syndrome (*N* = 8)	Endometriosis (*N* = 23)	Unexplained infertility (*N* = 7)	Other reasons (*N* = 4)	Total (*N* = 153)
Health parameters							
Age (years^†^)	32.6 ± 4.3	34.7 ± 5.0^b^	34.8 ± 3.2	31.8 ± 3.9	32.7 ± 2.9	36.0 ± 5.9	33.3 ± 4.5
Infertility^‡^							
Primary	42 (62.7%, 50.0–73.9)	15 (34.1%, 20.9–50.0)^c^	6 (75.0%, 35.6–95.5)	18 (78.3%, 55.8–91.7)	6 (85.7%, 42.0–99.2)^c^	2 (50.0%, 15.0–85.0)	89 (58.2%, 49.9–66.0)
Secondary	25 (37.3%, 26.1–50.0)	29 (65.9%, 50.0–79.1)^c^	2 (25.0%, 4.5–64.4)	5 (21.7%, 8.3–44.2)	1 (14.3%, 0.8–58.0)	2 (50.0%, 15.0–85.0)	64 (41.9%, 34.0–50.0)
Parity (N^†^)	0.3 ± 0.5	0.5 ± 0.7^b^	0.3 ± 0.5	0.1 ± 0.3	0.1 ± 0.4	0.5 ± 0.6	0.3 ± 0.6
S-FSH (U/L^†^)	7.6 ± 2.3	8.2 ± 3.2	7.0 ± 1.9	7.5 ± 2.7	6.9 ± 0.3	6.5 ± 2.2	7.7 ± 2.6
Smoking^‡^							
Never smoker	52 (78.8%, 66.7–87.5)	32 (76.2%, 60.2–87.4)	6 (75.0%, 35.6–95.5)	20 (87.0%, 65.3–96.6)	5 (71.4%, 30.3–94.9)	2 (66.7%, 12.5–98.2)	117 (77.5%, 69.8–83.7)
Past smoker	7 (10.6%, 4.7–21.2)	4 (9.5%, 3.1–23.51)	1 (12.5%, 0.7–53.3)	1 (4.3%, 0.2–24.0)^c^	1 (14.3%, 0.8–58.0)	1 (33.3%, 1.8–87.5)^c^	15 (9.9%, 5.9–16.1)
Current smoker	7 (10.6%, 4.7–21.2)	8 (19.0%, 9.1–34.6)	1 (12.5%, 0.7–53.3)	2 (8.7%, 1.5–29.5)	1 (14.3%, 0.8–58.0)	0	19 (12.6%, 7.9–19.2)
Treatment parameters^†^							
OPU S-E2 (pmol/L)	4497.1 ± 7950.9	2978.0 ± 1858.6	4195.5 ± 3117.8	3259.7 ± 1991.7	2960.0 ± 1723.2	2164.8 ± 1236.3	3711.8 ± 5429.4
OPU S-progesterone (nmol/L)	36.0 ± 22.5	26.1 ± 16.7^b^	34.2 ± 16.2	35.9 ± 17.0	40.3 ± 19.2	33.5 ± 26.9	33.3 ± 19.9
Total dose of FSH (IU)	1992.0 ± 704.0	2297.1 ± 925.0	1743.8 ± 525.6	1919.6 ± 726.3	2142.9 ± 574.2	2475.0 ± 656.7	2073.9 ± 702.8
Follicular diameter (mm)	21.4 ± 2.9	21.5 ± 3.3	21.7 ± 2.0^b^	19.9 ± 2.0	20.3 ± 1.6	19.3 ± 1.1^b^	21.1 ± 2.9
Oocytes (N)	11.6 ± 7.1	9.1 ± 5.5^b^	13.6 ± 9.3	11.1 ± 6.6	9.4 ± 8.3	11.3 ± 7.4	10.9 ± 6.8
Mature oocytes (N)^d^	9.2 ± 6.0	6.9 ± 4.5^b^	9.0 ± 7.8	9.6 ± 5.3	7.7 ± 6.8	8.5 ± 6.1	8.4 ± 5.7
2 PN-stage oocytes (N)	6.6 ± 5.0	4.6 ± 3.0^b^	6.0 ± 6.2	6.3 ± 4.0	4.7 ± 3.7	3.8 ± 4.1	5.8 ± 4.3
Good-quality embryos (N)^e^	3.8 ± 3.6	2.9 ± 2.3	2.9 ± 2.9	3.6 ± 3.1	2.4 ± 2.6	1.0 ± 2.0	3.3 ± 3.1
Rate of quality embryos (%)^f^	57.3 ± 30.0	56.4 ± 32.3	46.3 ± 38.4	52.9 ± 32.7	51.7 ± 33.6	14.8 ± 25.6	54.8 ± 31.6
Transferred embryos (N)	1.8 ± 0.5	1.8 ± 0.7	1.4 ± 0.7	1.9 ± 0.5	1.6 ± 0.8	1.3 ± 1.0	1.8 ± 0.6
Status of pregnancy^‡^							
hCG negative	44 (65.7%, 53.0–76.6)	29 (65.9%, 50.0–79.1)	6 (75.0%, 35.6–95.6)	16 (69.6%, 47.0–85.9)	5 (71.4%, 30.3–94.9)	3 (75.0%, 21.9–98.7)	103 (67.3%, 59.2–74.5)
Intrauterine	17 (25.4%, 15.9–37.7)	11 (25.0%, 13.7–40.6)	2 (25.0%, 4.5–64.4)	5 (21.7%, 8.3–44.2)	2 (28.6%, 5.1–69.7)	1 (25.0%, 1.3–78.1)	38 (24.8%, 18.4–32.6)
Biochemical	5 (7.5%, 2.8–17.3)	4 (9.1%, 3.0–22.6)	0	2 (8.7%, 1.5–29.5)	0	0	11 (7.2%, 3.8–12.8)
No ultrasound performed	1 (1.5%, 0.1–9.1)	0	0	0	0	0	1 (0.7%, 0.0–4.1)
Fetuses (N^†^)	0.3 ± 0.6	0.4 ± 0.7	0.3 ± 0.5	0.3 ± 0.6	0.3 ± 0.5	0.3 ± 0.5	0.3 ± 0.6

^†^Continuous variables are provided as mean ± standard deviation. ^‡^Categorical variables are provided as absolute numbers (percentage, 95% confidence interval of percentage). Differences between study groups: ^a^Reference group; ^b^ *t*-test, *P* < 0.05; ^c^Proportion test, *P* < 0.05. Associations between different parameters assessed by adjusted regression models are provided in the text. ^d^Number of oocytes which reached meiosis II stage at 4–6 h after oocyte retrieval. ^e^Number of embryos with at least four blastomeres and <20% fragmentation on the second day after-ICSI. ^f^The proportion (%) of good-quality embryos obtained from all 2PN fertilized oocytes. Abbreviations: hCG: human chorionic gonadotrophin; ICSI: intracytoplasmic sperm injection; OPU: oocyte pick-up day; PN: pronucleus; S-E2: serum estradiol; S-FSH: serum follicle-stimulating hormone.

**Table 3:** Biomarkers in the follicular fluid of patient groups.

	Male factor infertility (*N* = 67)^a^	Tubal factor infertility (*N* = 44)	Polycystic ovary syndrome (*N* = 8)	Endometriosis (*N* = 23)	Unexplained infertility (*N* = 7)	Other reasons (*N* = 4)	Total (*N* = 153)
Biomarkers (pg/mL)^†^							
G-CSF	82.5 (0–2464.0)	48.1 (0–4986.0)	104.1 (0–3156.0)	122.2 (0–4809.0)	118.5 (0–463.4)	23.7 (0–341.6)	89.7 (0–4986.0)
IL-1*β*	0 (0–236.8)	0 (0–53.6)	0 (0–29.0)	0 (0–110.3)	0 (0-0)	0 (0–143.1)	0 (0–236.8)
IL-6	0 (0–18.7)	0 (0–10.7)	0 (0–16.2)	0 (0–37.2)	0 (0-0)	0 (0–8.4)	0 (0–37.2)
IL-12p70	0 (0–24.9)	0 (0–6.1)	0 (0–8.1)	0 (0–21.0)	0 (0-0)^b^	0 (0–8.1)	0 (0–24.9)
IL-18	311.0 (0–722.0)	290.2 (0–812.5)	463.4 (0–648.5)	283.3 (44.6–874.3)	199.1 (0–255.5)^b^	310.9 (110.8–767.0)	297.2 (0–874.3)
IL-23	282.3 (0–1069.0)	208.7 (0–1280.0)	237.4 (0–746.4)	388.8 (0–1160.0)	408.5 (0–557.8)	120.3 (0–260.3)	260.3 (0–1280.0)
IFN-*α*	0 (0–150.7)	0 (0–107.6)	0 (0–93.5)	0 (0–114.2)	0 (0-0)	0 (0–161.9)	0 (0–161.9)
IFN-*γ*	0 (0–111.2)	0 (0–74.5)	0 (0–60.4)	0 (0–111.2)	0 (0-0)	9.5 (0–147.5)	9.5 (0–147.5)
TNF-*α*	0 (0–30.7)	0 (0–10.8)	0 (0–5.3)	0 (0–21.0)	0 (0-0)	0 (0–58.8)	0 (0–58.8)
IL-8	307.3 (119.4–4857.0)	367.2 (117.4–1117.0)^b^	417.6 (236.9–1032.0)	473.6 (172.8– 1879.0)^b^	424.3 (343.8–1472.0)	416.2 (172.8–2851.0)	371.2 (117.4–4857.0)
MCP-1	1019.0 (594.2–2046.0)	1054.0 (416.1–2564.0)	1067.0 (656.4–1572.0)	1033.0 (373.8–2780.0)	992.9 (818.1–1265.0)	801.7 (198.4–1598.0)^b^	1016.0 (198.4–2780.0)
MIP-1*α*	143.6 (0–5766.0)	80.6 (0–15990.0)	555.8 (0–19840.0)	227.6 (0–18230.0)	52.3 (0–3383.0)	136.3 (0–1788.0)	130.8 (0–19840.0)
MIP-1*β*	52.3 (6.0–1254.0)	48.2 (11.5–433.2)	38.7 (17.59–96.4)	51.7 (17.1–120.9)	40.7 (36.8–64.5)	63.5 (25.7–967.0)	48.4 (6.0–1254.0)
RANTES	97.4 (0–705.1)	97.4 (0–908.3)	50.1 (0–189.3)	146.6 (0–438.8)	77.4 (12.2–182.7)	74.1 (2.6–1428.0)	97.4 (0–1428.0)
sAPO-1/Fas	129.0 (0–564.2)	169.4 (0–9589.0)	152.1 (0–4469.0)	94.9 (0–520.4)	96.8 (64.3–292.5)	98.8 (0–226.8)	129.0 (0–9589.0)
CD44(var6)	8426.0 (5063.0–14030.0)	8554.0 (5348.0–20610.0)	10750.0 (6394.0–12130.0)^b^	8219.0 (5535.0–11770.0)	6619.0 (5168.0–9348.0)^b^	6836.0 (5611.0–10200.0)	8219.0 (5063.0–20610.0)

^†^Concentrations are provided as medians (minimum – maximum value). Differences between study groups: ^a^Reference group; ^b^Mann-Whitney *U*-test, *P* < 0.05. Associations between different parameters assessed by adjusted regression models are provided in the text.

**Table 4:** Relative* mRNA abundance of measured proteins in cumulus and mural granulosa cells.

Biomarkers	CGC ± SD	MGC ± SD	*P* value (paired *t*-test)
G-CSF	0.000216 ± 0.000151	0.000207 ± 0.000109	0.930
IL-1*β*	0.001025 ± 0.000640	0.070199 ± 0.108075	0.178
IL-6	0.000082 ± 0.000059	0.003981 ± 0.006412	0.209
IL-12A	0.000104 ± 0.000047	0.000209 ± 0.000083	0.022
IL-18	0.001506 ± 0.000958	0.007472 ± 0.002346	< 0.001
IL-23A	0.000009 ± 0.000004	0.000025 ± 0.000014	0.016
IFN-*α*	0.000236 ± 0.000186	0.000044 ± 0.000021	0.127
IFN-*γ*	0.000064 ± 0.000045	0.000027 ± 0.000021	0.157
TNF-*α*	0.000133 ± 0.000150	0.001899 ± 0.002363	0.117
IL-8	0.022214 ± 0.009590	0.487650 ± 0.431439	0.045
MCP-1	0.003409 ± 0.004521	0.008618 ± 0.009842	0.239
MIP-1*α*	0.000015 ± 0.000011	0.000451 ± 0.000718	0.191
MIP-1*β*	0.001029 ± 0.000864	0.055073 ± 0.071566	0.122
RANTES	0.000650 ± 0.000282	0.014258 ± 0.021169	0.293
sAPO-1/FAS	0.000024 ± 0.000014	0.000024 ± 0.000016	0.979
CD44(v6)	0.000024 ± 0.000006	0.000045 ± 0.000028	0.158

*As compared to the average of three housekeeping gene transcripts: beta actin, glyceraldehyde-3-phosphate dehydrogenase, and ribosomal protein RPL13A. Abbreviations: CGC: cumulus granulosa cells; MGC: mural granulosa cells; SD: standard deviation.
